# A Simple Risk Score for Identifying Individuals with Impaired Fasting Glucose in the Southern Chinese Population

**DOI:** 10.3390/ijerph120201237

**Published:** 2015-01-23

**Authors:** Hui Wang, Tao Liu, Quan Qiu, Peng Ding, Yan-Hui He, Wei-Qing Chen

**Affiliations:** 1 Guangdong Provincial Key Laboratory of Food, Nutrition and Health, Department of Biostatistics and Epidemiology, School of Public Health, Sun Yat-Sen University, Guangzhou 510080, China; E-Mails: wanghui-78@163.com (H.W.); gztt_2002@163.com (T.L.); qiuquan2008s@126.com (Q.Q.); ben_1234567@hotmail.com (P.D.); hyhhappy2003@163.com (Y.-H.H.); 2 Department of Epidemiology and Biostatistics and Guangdong Key Lab of Molecular Epidemiology, School of Public Health, Guangdong Pharmaceutical University, Guangzhou 510006, China

**Keywords:** risk assessment, score, impaired fast glucose, Southern Chinese

## Abstract

This study aimed to develop and validate a simple risk score for detecting individuals with impaired fasting glucose (IFG) among the Southern Chinese population. A sample of participants aged ≥20 years and without known diabetes from the 2006–2007 Guangzhou diabetes cross-sectional survey was used to develop separate risk scores for men and women. The participants completed a self-administered structured questionnaire and underwent simple clinical measurements. The risk scores were developed by multiple logistic regression analysis. External validation was performed based on three other studies: the 2007 Zhuhai rural population-based study, the 2008–2010 Guangzhou diabetes cross-sectional study and the 2007 Tibet population-based study. Performance of the scores was measured with the Hosmer-Lemeshow goodness-of-fit test and ROC *c*-statistic. Age, waist circumference, body mass index and family history of diabetes were included in the risk score for both men and women, with the additional factor of hypertension for men. The ROC c-statistic was 0.70 for both men and women in the derivation samples. Risk scores of ≥28 for men and ≥18 for women showed respective sensitivity, specificity, positive predictive value and negative predictive value of 56.6%, 71.7%, 13.0% and 96.0% for men and 68.7%, 60.2%, 11% and 96.0% for women in the derivation population. The scores performed comparably with the Zhuhai rural sample and the 2008–2010 Guangzhou urban samples but poorly in the Tibet sample. The performance of pre-existing USA, Shanghai, and Chengdu risk scores was poorer in our population than in their original study populations. The results suggest that the developed simple IFG risk scores can be generalized in Guangzhou city and nearby rural regions and may help primary health care workers to identify individuals with IFG in their practice.

## 1. Introduction

Pre-diabetes, in which blood glucose concentrations are higher than normal but have not yet met the absolute definition of diabetes, is usually called borderline diabetes and represents a high-risk state for the development of diabetes [1]. Pre-diabetes includes impaired fasting glucose (IFG) and impaired glucose tolerance (IGT). The prevalence of pre-diabetes has rapidly increased in developing countries over the past years [2] and is much higher even than that of type 2 diabetes (T2DM) [3]. Pre-diabetes has high probability to progress to T2DM [3] and is also associated with an increased risk of mortality and macrovascular and microvascular diseases [4]. Many studies have shown that fortunately, lifestyle or medication interventions at the pre-diabetes stage can delay or prevent the progression of pre-diabetes to T2DM [5,6,7,8,9,10]. However, pre-diabetes is asymptomatic, and the majority of individuals with this condition remain undiagnosed and untreated [2]. Therefore, an important step in delaying or preventing T2DM and its associated complications is to identify individuals with pre-diabetes in the population as early as possible so that they can be treated appropriately.

In past decades, fasting plasma glucose (FPG) and the oral glucose tolerance test (OGTT) were used to screen those with pre-diabetes and undiagnosed T2DM in the population. However, these two methods are unlikely to remain a feasible strategy for tackling the rapidly rising prevalence of pre-diabetes due to their complexity, high cost and invasiveness. Currently, many self-administered risk scores are available to assess undiagnosed diabetes in different countries and regions and for different races [11,12,13,14,15,16,17,18,19,20,21,22,23]. The self-administered risk score has many advantages compared with FPG and the OGTT; for example, it is simple, cheap, noninvasive and easily accepted. Presently, to the best of our knowledge, few studies have addressed the establishment of risk scores especially to detect pre-diabetes. Hence, it is necessary to establish such a score that will contribute to diabetic screening and prevention in the Southern Chinese population. The current study aimed to develop a risk score to identify individuals with IFG (a form of pre-diabetes) in the Southern Chinese population and validate the risk score in three other population-based samples. We further compared the performance of our risk score with that of several published risk scores for detecting individuals with IFG in our study population.

## 2. Methods

### 2.1. Population for Establishing the Risk Scores

The derivation sample of the present study came from the 2006–2007 Guangzhou diabetes cross-sectional population-based survey in urban communities. As described previously [24], multiple-stage random cluster sampling was performed to recruit the study subjects. All residents aged ≥20 years who had lived in Guangzhou for at least 5 years were invited, of whom 6197 took part. We discovered 620 subjects with diagnosed or undiagnosed diabetes before we performed the survey, who were excluded from the study sample.

### 2.2. Population for Validating the Risk Scores

Internal validation of the scores was performed in the derivation sample, and three validation samples were used for external validation. Validation sample 1 was based on the 2007 Zhuhai population-based survey. This survey targeted the rural population of Zhuhai, of whom 1186 individuals took part and 87 individuals with diagnosed or undiagnosed diabetes before the survey were excluded. Validation sample 2 came from the 2008–2010 Guangzhou cross-sectional study on diabetes in urban communities, from which 3162 subjects were recruited and 174 with diabetes were excluded. The study set for validation sample 3 was the 2007 Tibet population-based study with 1289 Tibetan individuals participating [25], of whom 30 with diabetes were excluded. The study design and data collection of the three studies were almost the same as those of the study for the derivation sample. We also used three other risk assessment scores [26,27,28] and validated them in our derivation population. All of the studies were approved by the ethics committee of Sun Yat-Sen University, and informed consent was obtained from each participant before starting the data collection.

### 2.3. Data Collection

Data on demographic characteristics and health-related behaviors were collected through a self-administered structured questionnaire, and a standardized physical examination (blood pressure, height, weight and waist circumference) was performed on each participant. A venous blood sample was collected in the morning after an overnight fast to measure FPG. All of the above data were collected at local community health care centers.

The related factors are defined as follows: age, body mass index (BMI) and waist circumference were included as continuous variables in the univariate analysis or in the initial models for determination of score performance, and then as fractiles in the final models. Age was divided into four groups: 20–39 years, 40–49 years, 50–59 years and ≥60 years. According to the study of China Obesity Task Force [29], BMI was divided into three groups: <24 kg/m^2^, 24 kg/m^2^ to 28 kg/m^2^ and ≥28 kg/m^2^. On the basis of the central obesity definitions of the International Diabetes Foundation for Asians [30], waist circumference was divided into two groups: <90 cm and ≥90 cm for men and <80 cm and ≥80 cm for women. Hypertension was defined as a systolic blood pressure ≥140 mmHg, diastolic blood pressure ≥90 mmHg or diagnosed hypertension and taking antihypertensive drugs for more than 2 weeks [31]. Family history of diabetes was defined as a history of diabetes in first-degree relatives (parents, siblings and children) at any age.

### 2.4. Diagnostic Criteria for Impaired Fasting Glucose (IFG)

According to the 1999 WHO diagnostic criteria [32], IFG is defined as a fasting plasma glucose concentration from ≥6.1 mmol/L to <7.0 mmol/L.

### 2.5. Statistical Analysis

The risk scores were derived from the data drawn from the 2006–2007 Guangzhou survey. Candidate prediction variables were selected by univariate logistic regression analysis. Proposed variables were retained if they achieved the set criterion of *p* ≤ 0.05 for statistical significance. Considering the varying contributions of risk factors for IFG across sex, we developed the scores for men and women separately using multivariate logistic regression. We added the retained candidate variables (not including the laboratory-tested ones) into the equations one by one from highest to lowest odds ratios according to their values in the univariate analysis. To keep the risk scores simple and easy to use, interaction terms between the independent variables were not considered. The Hosmer-Lemeshow goodness of fit test was performed to determine whether the addition of a variable to a model improved the calibration of the model (the extent of matching between predicted and observed risk of IFG), and the receiver operating characteristic (ROC) *c*-statistic (equivalent to the area under the curve (AUC)) was used to assess whether the addition of a variable to a model improved the discrimination of the model (the ability to stratify high risk or non-high risk for IFG). The point values of the IFG risk scores were derived by multiplying β, which was derived from the final multivariate regression model, by the constant 10 and were rounded to the nearest integer for ease of use. The higher the score, the greater is the risk of developing IFG.

Cut-off points were used to assess whether individuals are at risk of IFG. with the point beyond the cut-off indicating a high risk of developing IFG. As a non-invasive risk score, our risk score, would be used in the first step of a screening program, so sensitivity should weigh higher than specificity. Consequently, the optimal cut-off points of the risk scores for clinical decision-making in general practice were determined as a minimum level of sensitivity (75%) instead of using the largest Youden Index. We also evaluated positive predictive value (PPV) and negative predictive value (NPV) based on the different diabetic prevalence of the different samples. The comparison of ROC-AUCs was performed by *Z*-test. SPSS 13.0 was used for all statistical analyses.

## 3. Results

### 3.1. Characteristics of the Study Populations

There were 6197 eligible participants in the derivation sample, and the response rate was 95%. Of these participants, 2094 (33.79%) were men and 4103 (66.21%) were women. The prevalence of IFG among the men (6.9%) was slightly higher than that among the women (6.6%). Among the patients with IFG, the men had a higher prevalence rate of hypertension (55.6% *vs.* 46.8%) and a lower prevalence of central obesity (28.6% *vs.* 55.6%) versus those of the women.

Among all the samples, compared with the participants in the Guangzhou and Zhuhai studies, those in the Tibet study had had a lower prevalence rate of diabetes, IFG and family history of diabetes but had a higher prevalence rate of obesity. The characteristics of the participants are shown in Table 1.

**Table 1:** Baseline characteristics of the four different study samples.

Variable or Statistic	Derivation Sample	Validation Sample 1	Validation Sample 2	Validation Sample 3	*P* ^a^ Value
N (% of men)	6033 (32.0)	1186 (37.8)	3162 (28.4)	1289 (28.4)	--
Mean age (year)	51.6 ± 12.7	49.4 ± 13.2	57.5 ± 5.2	43.6 ± 14.3	<0.001
BMI(kg/m^2^)	23.5 ± 3.4	23.0 ± 3.3	23.3 ± 3.2	24.2 ± 4.0	0.04
Waist circumference(cm)	79.1 ± 9.4	77.7 ± 9.3	82.4 ± 9.1	82.4 ± 12.1	<0.001
Systolic blood pressure (mmHg)	123.4 ± 19.7	128.6 ± 20.8	123.6 ± 17.7	120.5 ± 22.9	0.002
Diastolic blood pressure (mmHg)	79.1 ± 10.6	81.6 ± 10.3	78.2 ± 10.7	82.4 ± 14.0	0.01
Fast blood glucose (mmol/L)	5.54 ± 1.49	5.63 ± 1.52	4.77 ± 1.46	4.92 ± 1.35	0.03
Number of patients with IFG	384	106	95	37	--
IFG (%)	6.2	8.9	3.0	2.9	0.02
Obesity (%) ^b^	9.0	6.9	7.1	16.9	0.01
Central obesity (%) ^c^	32.4	34.6	41.6	45.5	0.02
Hypertension (%)	32.8	33.7	34.3	30.3	0.33
Family history of diabetes (%)	17.6	6.1	16.3	2.2	<0.001

Data are means ± SD or percentages. BMI: body mass index; HDL-C: high-density lipoprotein cholesterol; LDL-C: low-density lipoprotein cholesterol. Derivation sample: the Guangzhou urban sample in the year 2006–2007, including subjects above 20 years old; Validation sample 1: the Zhuhai rural sample, including subjects above 20 years old; Validation sample 2: the Guangzhou sample in the year 2008–2010, including subjects above 40 years old; Validation sample 3: the Tibet sample, including subjects above 20 years old. **^a^** tested by Chi-square or one-way ANOVA. **^b^** Defined as BMI ≥ 28. **^c^** Defined as a waist circumference measurement ≥90 cm for men or ≥80 cm for women.

### 3.2. Development of the Risk Scores

After univariate logistic regression analysis, the candidate prediction variables for the risk scores were age, BMI, waist circumference, systolic blood pressure, diastolic blood pressure and family history of diabetes. The model prediction for both men and women improved significantly (*p* < 0.05) with the addition of the age, BMI, waist circumference and family history of diabetes variables into the equations. Adding the variable of hypertension improved the model prediction significantly for men but not for women. When we added the above variables, which contributed to the equations (scores) one by one, the ROC c-statistic improved slightly each time (results not shown). The formulas for the prediction models for men and for women are as follows, with age, waist circumference and BMI regarded as continuous variables:

(1) $$\begin{array}{l} ln\left[p/\left(1-p\right)\right]for\;men\;=\;-8.887\;+\;0.04\;\times \;Age\;+\;0.02\;\times \;Waist\;circumference\;+\;0.09\;\times \;BMI\;+\;\\ \;0.25\;\times \;Family\;history\;of\;diabetes\;+\;0.15\;\times \;Hypertension \end{array}$$

and:

(2) $$\begin{array}{l} ln\left[p/\left(1-p\right)\right]for\;women\;=\;-8.918\;+\;0.04\;\times \;Age\;+\;0.05\;\times \;Waist\;circumference\;+\;0.008\;\times \;BMI\;+\;\\ \;0.45\;\times \;Family\;history\;of\;diabetes \end{array}$$

The final models and the point values of the risk scores are shown in Table 2. The scores were the point values ranging between 0 to the maximum scores in Table 2.

**Table 2:** β-coefficient, ORs and risk scores of predictors in the models for detecting IFG based on the derivation sample.

Variable or Statistic	Men			Women		
	β Coefficient	OR (95% CI)	Score	β Coefficient	OR (95% CI)	Score
Age(years): 20–39	--	1.00	0	--	1.00	0
40–49	1.77	5.85 (1.68–20.34)	18	1.10	3.00 (1.53–5.90)	11
50–59	1.95	6.99 (2.12–23.03)	19	1.53	4.59 (2.42–8.72)	15
Over 60	2.04	7.69 (2.33–25.40)	20	1.95	7.03 (3.68–13.41)	20
Waist circumference(cm): men <90,women <80	--	1.00	0	--	1.00	0
men ≥90,women ≥80	−0.12	0.89 (0.55–1.43)	−1	0.54	1.72 (1.20–2.48)	5
Family history of diabetes:						
No	--	1.00	0	--	1.00	0
Yes	0.16	1.18 (0.69–2.01)	2	0.46	1.58 (1.12–2.22)	5
BMI: BMI < 24	--	1.00	0	--	1.00	0
24 ≤ BMI < 28	0.44	1.56 (0.93–2.61)	4	0.09	1.09 (0.76–1.58)	1
BMI ≥ 28	0.93	2.54 (1.33–4.86)	9	0.49	1.63 (1.00–2.64)	5
Hypertension: No	--	1.00	0	--	--	--
Yes	0.78	2.19 (1.43–3.35)	8	--	--	--
Maximum score			38			35

BMI: body mass index.

### 3.3. Internal and External Validation of the Risk Scores

**Figure 1:**
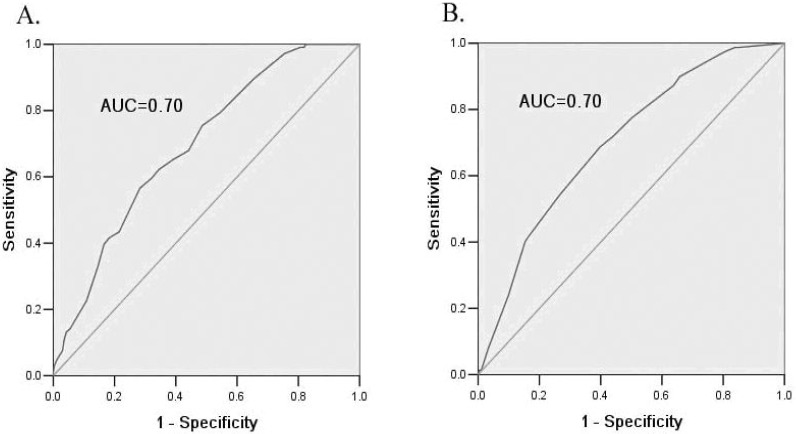

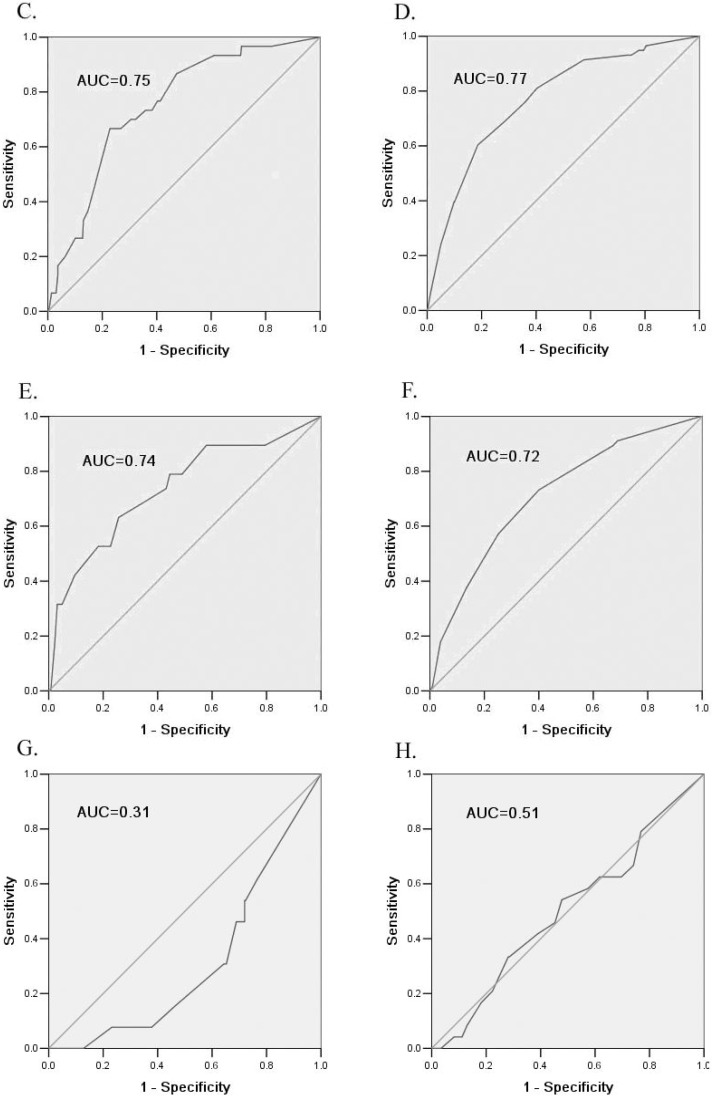
ROC curves of the risk scores for detecting IFG. (**A**). ROC curve for men in the derivation sample; (**B**). ROC curve for women in the derivation sample; (**C**). ROC curve for men in the validation sample 1; (**D**). ROC curve for women in the validation sample 1; (**E**). ROC curve for men in the validation sample 2; (**F**). ROC curve for women in the validation sample 2; (**G**). ROC curve for men in the validation sample 3; (**H**). ROC curve for women in the validation sample 3.

For the internal validation of the risk scores for both men and women, the *p* values of the Hosmer-Lemeshow goodness-of-fit tests were 0.40 and 0.38, respectively, and the values of the ROC c-statistic were 0.70 for both sexes (Table 3 and Figure 1). We also calculated the ROCs of single anthropometrics variables for predicting IFG. The values of the ROCs of the single BMI and waist circumference variables were 0.64 and 0.67, respectively, for women, and 0.61 for both for men, and all were lower than those of the risk scores.

**Table 3:** Internal and external validation studies of the different models.

Validation	Model for Men	Model for Women
Internal validation studies in the derivation sample		
Goodness of fit(*P* value)	0.40	0.38
ROC *c*-statistic(95% CI)	0.70 (0.65–0.74)	0.70 (0.67–0.73)
External validation studies in the validation sample 1		
Goodness of fit(*P* value)	0.59	0.96
ROC *c*-statistic(95% CI)	0.75 (0.67–0.83)	0.77 (0.71–0.83)
External validation studies in the validation sample 2		
Goodness of fit(*P* value)	0.78	0.56
ROC *c*-statistic(95% CI)	0.74 (0.61–0.86)	0.72 (0.65–0.78)
External validation studies in the validation sample 3		
Goodness of fit(*P* value)	0.49	0.54
ROC c-statistic(95% CI)	0.31 (0.20–0.43)	0.50 (0.38–0.61)

BMI: body mass index; WC: waist circumference. Derivation sample: the 2006–2007 Guangzhou urban sample; Validation sample 1: the Zhuhai rural sample; Validation sample 2: the 2008–2010 Guangzhou urban sample; Validation sample 3: the Tibet sample. Goodness of fit was tested by Hosmer-Lemeshow test.

The optimal cut-off points of the risk scores were 23 for men and 16 for women. These cut-off points defined approximately 50.5% and 52.1% of the derivation population as having IFG for the men and women, respectively. Sensitivity, specificity, PPV and NPV were 75.5%, 51.4%, 12.0% and 97.0% for men and 77.5%, 49.8%, 10% and 97.0% for women, respectively, in the derivation population (Table 4). In the external validation, the risk scores performed well in the Zhuhai rural sample and the 2008–2010 Guangzhou urban sample: The *p* values of the Hosmer-Lemeshow goodness-of-fit tests were between 0.56 and 0.96, and the values of the ROC c-statistic were from 0.72 to 0.77 (Table 3 and Figure 1). However, the values of the ROC c-statistic of the risk scores in the Tibet sample were 0.31 for men and 0.51 for women, respectively. Sensitivity, specificity, PPV and NPV at the optimal cut-off points of risk scores (23 for men and 16 for women) in the three validation samples are presented in Table 4. Compared with the performance in the derivation sample, the three validation samples had similar specificity and NPV. Validation sample 1 (Zhuhai rural sample) had a much higher PPV than the other validation samples. Although the PPV of validation sample 2 (2008–2010 Guangzhou sample) was lower than that of the derivation sample, the NPV was higher. However, both the sensitivity and specificity of validation sample 3 (the Tibet sample) were less than those of the other samples.

**Table 4:** The performance of the risk score at cut-off points for detecting IFG in the derivation sample and validation samples.

Total Score	Number (%)	Sensitivity(%)	Specificity (%)	PPV (%)	NPV (%)
Derivation sample					
Model for men					
≥23	747 (50.5)	75.5	51.4	12	97
Model for women					
≥16	1755 (52.1)	77.5	49.8	10	97
Validation sample 1					
Men (≥23)	136 (41.5)	73.3	64.1	13	97
Women (≥16)	255 (44.4)	81.0	59.7	19	96
Validation sample 2					
Men (≥23)	430 (51.7)	78.9	51.0	6	99
Women (≥16)	1254 (58.1)	89.3	41.8	5	99
Validation sample 3					
Men (≥23)	160 (41.1)	30.8	48.8	2	96
Women (≥16)	362 (40.5)	41.7	59.0	4	96

PPV: positive predictive value; NPV: negative predictive value; Derivation sample: The 2006–2007 Guangzhou urban sample. Validation sample 1: the Zhuhai rural sample; Validation sample 2: the 2008–2010 Guangzhou urban sample; Validation sample 3: the Tibet sample.

### 3.4. Comparison of the Current Risk Scores with Other Existing Scores for Pre-Diabetes

The AUCs of the risk scores in the USA [26], Chengdu [27] and Shanghai [28] original populations were similar to those our scores, but performance of the scores was poorer in our population than in the original study populations, which can be seen by comparison with their AUCs (0.66 *vs.* 0.74 for the USA score; 0.67 *vs.* 0.70 for the Shanghai score; and 0.66 *vs.* 0.72 for men and 0.67 *vs.* 0.73 for women for the Chengdu score) (Table 5).

**Table 5:** Other pre-diabetes risk scores developed in other populations and performances in the current derivation study population.

Derivation Population (Publication Year)	Predictors Involved	Optimal Cut-Off Value (Range)		Area under the (95%CI)				Sensitivity at the Optimal Cut-Off Value (%)		Specificity at the Optimal Cut-off Value (%)		
				In Original Population	In the Population of This Study	*p* Value ^*^	In Original Population	In the Population of This Study	In Original Population		In the Population of This Study
USA (2008)	Age, sex, BMI, hypertension, family history of diabetes, resting heart rate	5 (0–16)		0.74	0.66 (0.63–0.68)	0.04	87.0	92.0		43.3	26.4
Shanghai, China (2009)	Age, waist circumference, family history of diabetes, systolic blood pressure	5 (4–11.7)		0.70	0.67 (0.64–0.70)	0.06	68.2	68.5		61.7	54.9
Chengdu, China (2010)	Age, occupational physical activity, family history of diabetes, BMI, central obesity, hypertension, leisure physical activity, gestational diabetes, number of deliveries	Men: 5 (0–18)		Men: 0.72 (0.69–0.74)	Men: 0.66 (0.61–0.72)	0.06	Men: 74.1	Men: 73.3		Men: 58.4	Men: 54.2
		Women: 6 (0–22)		Women: 0.73 (0.71–0.75)	Women: 0.67 (0.63–0.71)		Women: 75.6	Women: 44.5		Women: 65.6	Women: 76.1

^*^ compared with the AUC of the IFG risk scores in our study.

## 4. Discussion

Using data from a community-based cross-sectional survey on T2DM in adult populations from Guangzhou, China [24], we developed a simple risk score for detecting IFG among men or women in this particular population. Factors included in the risk score for both sexes were age, family history of diabetes, waist circumference and BMI for women, with the additional variable of hypertension for men. Internal validation proved that the scores were sensitive and specific for detecting IFG, and further external validation showed that the scores had good overall performance, good calibration and good discrimination in the investigated Southern Chinese population.

To date, a number of risk scores for detecting individuals with undiagnosed T2DM (or pre-diabetes included) have been developed for different countries and different races [13,14,15,18,20,21,22,23,33,34,35]. Most were established for individuals aged over 35 years old [13,15,21,22,23,33,34,35]. For example, a quick self-assessment tool was developed in a Chinese population aged 35–74 years to identify individuals at high risk of type 2 diabetes [23], and a risk score for predicting incident diabetes in a Thai population was developed for subjects aged 35–55 years [15]. However, given the increasing burden of T2DM among youthful populations, we expect IFG to occur at even younger ages. As we expected, the prediction models for undiagnosed diabetes in our previous study are weighted more toward age and waist circumference, and less toward BMI, than the prediction models for pre-diabetes in this study, so the existing models for predicting the risk of developing diabetes may not be especially suitable for individuals with IFG. Accordingly, the establishment of specific risk scores to identify individuals at high risk of pre-diabetes is necessary.

Similar to the risk scores for undiagnosed diabetes that we developed previously, we designed the models in this study to use only those factors that can be either self-reported or easily measured. Further, we developed separate risk scores for detecting IFG in men and women. All of the risk factors in our scores are easily obtained by asking several questions or by anthropometric measurements, thus allowing a simple, inexpensive, quick and noninvasive process. Therefore, our risk scores may provide primary health care workers with a tool to assess their patient’s risk of IFG using the patients’ health records. The scores also have potential application as web-based screening tools to improve health awareness and to encourage compliance with physician-recommended lifestyle changes.

Internal and external validation proved the good overall performance of our scores in the Southern Chinese population investigated. The AUCs of our risk scores for both men and women (0.70) were similar to the AUCs of risk scores developed in the USA (0.74) [26] and in Chengdu, a provincial capital city in Western China (0.72 for men and 0.73 for women) [27], indicating that our risk scores appeared to perform comparably with existing risk scores using quantitative criteria, although small differences were present.

On the basis of our scores, a man with a score of ≥23 or a woman with a score of ≥16 would be regarded as being at risk of IFG and would be advised to undergo additional testing. The sensitivity and specificity at these two scores were 75.5% and 51.4% in men 77.5% and 49.8% in women, respectively, which varied slightly from those of the pre-diabetes risk scores for the USA residents (87.0% and 43.3%) [26], Shanghai urban residents (68.2% and 61.7%) [28] and Chengdu residents (74.1% and 58.4% for men; 75.6% and 65.6% for women) [27]. This may be due to the different performances between these risk scores.

Performance of our risk scores developed in the southern Chinese population investigated proved to be poor in a population in Tibet (a high-altitude region of Southwest China) through external validation. This result again confirmed that specific risk scores must be developed for different populations in different areas, as shown in previous studies on the development of risk scores to identify individuals with undiagnosed diabetes [36,37]. For instance, a study by Ramachandran *et al.* [20] proved that a diabetes risk score developed for a native Asian Indian population could not be generalized to South Asian residents in the UK. Another study by Glümer *et al.* [36] showed that the risk scores for undiagnosed T2DM developed and validated in Caucasians performed similarly in other Caucasian populations but poorly in non-Caucasian populations. Furthermore, we used our derivation population to validate three existing pre-diabetes risk assessment scores and compared the AUCs between them. The AUCs of the scores for the USA [26], Chengdu [27] and Shanghai [28] original populations were similar to those of our scores, but the scores showed poorer performance in our population than in their original populations. One possible explanation may be that genetic and environmental determinants for T2DM or pre-diabetes may differ between different ethnic groups. The people in validation sample 3 are Tibetans who live at an altitude of more than 3600 m in Southwest China and have a different genetic background, diet, lifestyle and climate from the derivation sample of Cantonese in Guangzhou. Another possible reason is that the distribution of risk factors for populations of the same ethnicity living in a different cultural context, such as different body size, diet, lifestyle and climate, may be different. Our derivation sample, validation sample 1 and validation sample 2 comprise people of Cantonese ethnicity (a branch of Han Chinese) living in the Pearl River Delta region in the southern areas of China who share a similar diet, lifestyle and culture. However, Han Chinese in Shanghai and Chengdu live in eastern or southwestern areas of China, respectively, and compared with the Cantonese, they tend to have a larger body frame and their diet consists of more sweet or spicy foods. Summarily, our risk scores derived from the Guangzhou population are suitable only for people living in the Pearl River Delta region in Southern China and not for all Chinese.

There are several advantages of the present study. First, the risk scores are very simple and relatively easy to interpret, so they can be used by general practitioners in poorer areas and even by the general population. Second, the derivation sample was from a community population-based study, so representation was good. Quality control in this study was good and included strict training of investigators, uniform protocols, face-to-face investigations, standardized tests and severe re-checking rules. Third, we used three independent validation samples comprising varied populations in Southern China, Guangzhou and Tibet. Finally, the sample size was adequate to achieve good statistical power and stability of the developed scores.

The present study also has several limitations. First, it examined only FPG as an outcome. Although there is substantial overlap between impaired fasting glucose and impaired glucose tolerance, we did not address impaired glucose tolerance, which would require an OGTT. When considering probable compliance rates of the OGTT, however, using FPG levels may be the best initial strategy to screen for pre-diabetes [38]. Second, the risk scores for detecting IFG developed in the present study were based on cross-sectional data, and therefore, they can only be used to identify prevalent cases of IFG rather than incident cases of IFG.

Additionally, in primary care settings, practitioners should consider two aspects. First, because the PPV and NPV of the proposed risk score will vary based on the prevalence of IFG in the populations tested, practitioners should use this IFG prediction tool according to the actual conditions. Second, the risk scores have high sensitivity and high NPV. Therefore, practitioners should inform their patients with scores above the cut-off values that they have a potential risk of developing diabetes and should change their unhealthy lifestyles to reduce the risk of the onset of diabetes.

## 5. Conclusions

We have developed two simple risk scores that can be used to screen for IFG separately in Southern Chinese men and women and have done so using only routinely collected information with no laboratory tests required. A subject with a score above the cut-off value has a potential risk of developing diabetes and should be advised to change his/her unhealthy lifestyle to reduce the risk of the onset of diabetes. Internal and external validation showed that the scores performed well. Our results suggest that the risk scores could be used to detect IFG in the Southern Chinese population or a population in which the distribution of risk factors and their association with prevalent IFG are similar.
